# The mechanisms of MicroRNA 21 in premature ovarian insufficiency mice with mesenchymal stem cells transplantation: The involved molecular and immunological mechanisms

**DOI:** 10.1186/s13048-024-01390-8

**Published:** 2024-04-04

**Authors:** Na Yin, Chao Luo, Lun Wei, Guangzhao Yang, Le Bo, Caiping Mao

**Affiliations:** 1 Reproductive Medicine Center, First Affiliated Hospital of Soochow University, 899 Pinghai Rd, Suzhou, 215000 Jiangsu China; 2 International Peace Maternity and Child Health Hospital of China Welfare Institute, Shanghai, 200030 China

**Keywords:** MicroRNA 21, Umbilical cord-derived mesenchymal stem cells, Premature ovarian insufficiency, PTEN, CD8^+^CD28^−^T cells

## Abstract

Umbilical cord-derived mesenchymal stem cell (UCMSC) transplantation has been deeply explored for premature ovarian insufficiency (POI) disease. However, the associated mechanism remains to be researched. To explore whether and how the microRNA 21 (miR-21) functions in POI mice with UCMSCs transplantation, the autoimmune-induced POI mice model was built up, transplanted with or without UCMSCs transfect with the LV-hsa-miR-21-5p/LV-hsa-miR-21-5p-inhibition, with the transfection efficiency analyzed by QRT-PCR. Mice hormone secretion and the anti-Zona pellucida antibody (AZPAb) levels were analyzed, the ovarian morphological changes and folliculogenesis were observed, and the ovarian apoptosis cells were detected to evaluate ovarian function. The expression and localization of the PTEN/Akt/FOXO3a signal pathway-related cytokines were analyzed in mice ovaries.

Additionally, the spleen levels of CD8 + CD28-T cells were tested and qualified with its significant secretory factor, interleukin 10 (IL-10). We found that with the LV-hsa-miR-21-5p-inhibition-UCMSCs transplantation, the mice ovarian function can be hardly recovered than mice with LV-NC-UCMSCs transplantation, and the PTEN/Akt/FOXO3a signal pathway was activated. The expression levels of the CD8 + CD28-T cells were decreased, with the decreased levels of the IL-10 expression. In contrast, in mice with the LV-hsa-miR-21-5p-UCMSCs transplantation, the injured ovarian function can be reversed, and the PTEN/AKT/FOXO3a signal pathway was detected activated, with the increased levels of the CD8 + CD28-T cells, and the increased serum levels of IL-10. In conclusion, miR-21 improves the ovarian function recovery of POI mice with UCMSCs transplantation, and the mechanisms may be through suppressing the PTEN/AKT/FOXO3a signal pathway and up-regulating the circulating of the CD8 + CD28-T cells.

**Impact statement**

As a heterogeneous disorder, premature ovarian insufficiency (POI) disease can cause menopausal syndrome and infertility, which tortured more and more women in less than 40 years. However, a radical cure remains evaluated. Umbilical cord-derived mesenchymal stem cell (UCMSC) transplantation has been deeply explored for POI disease, while the therapeutic efficiency and stability remain to be improved. This experiment investigates the role of microRNA21 in treating POI mice with UCMSCs transplantation. Further, it unravels the underlying molecular and immunological mechanisms, which provided strategies for clinical that changing some important cytokines in UCMSCs may increase the stability of MSCs’ function during the therapy process. The research above Studying the underlying mechanisms of the therapeutic process may help more women have their babies and further maintain harmonious family relations.

## Introduction

As a heterogeneous disorder, premature ovarian insufficiency (POI) is characterized by elevated gonadotropins and descended estrogen levels, which may cause menopausal syndrome and infertility and are prevalent in 1-3% of women less than 40 years old [1]. There is no denying that genetic, autoimmune, environmental, and idiopathic factors are closely associated with the disease, even if the exact pathogenesis remains to be explored [2]. Among the factors above, the autoimmune factors may affect 4–30% of women with POI disorder, and the ovarian dysfunction may be related to zona pellucida 3 (ZP3) antigens, which is an acellular matrix surrounding the developing and ovulated oocytes and is functioned as the significant sperm receptor in fertilization [3]. Antibodies to ZP antigen (Antizona pellucida antibodies, AZPAb) may interfere with the sperm-oocyte interaction, leading to follicular depletion and amenorrhea [4]. Therefore, the ZP3-induced POI mice model was built and investigated in this study.

Unfortunately, the POI disorder tortured more and more women, and a radical cure remains evaluated. In recent years, the transplantation of mesenchymal stem cells (MSCs) has been considered to be an effective therapy for treating POI disorder [1]. The mechanism of treating POI with MSCs can be summarized as follows: ①MSCs have a “homing” effect [5]; ②MSCs can promote the growth and development of follicles at all developmental stages [6]; ③MSCs may induce and differentiate into primordial germ cells (uncertain); ④MSCs can directly differentiate into GCs or inhibit the apoptosis of GCs [2]; ⑤MSCs can promote the formation of ovarian blood vessels [7]; ⑥MSCs have immunomodulatory and anti-inflammatory effects [8] and ⑦MSCs can reduce oxidative stress [9, 10]. However, the mechanisms above still need to be explored, and more in-depth laboratory experiments are still necessary to solve this scientific problem. With the characteristics of vast sources, easy extraction, and low immunogenicity, human umbilical cord-derived mesenchymal stem cells (UCMSCs) have been recognized as the preferred MSCs for transplantation [11], which have been successfully applied into clinical [12].In recent years, gene therapy associated with the regulation of microRNAs (miRNAs) is getting more and more attention in all domains but MSC therapy [13].

MiRNAs are recognized to regulate cell proliferation, differentiation, and cell cycle [14], which are also involved in the development and differentiation of immune cells or the regulation of the immune response [15]. MiR-21 is highly expressed in various cells and tissues [16], promoting cell proliferation and inhibiting apoptosis. It is decisive in regulating granulosa cells (GCs) apoptosis and follicular development [17]. It is reported to be manifested through suppressing critical apoptotic genes such as phosphatase and tensin homolog (PTEN) [18]. Much research has found that the balance of phosphatidylinositol 3-kinase (PI3K) signaling plays a vital role in the primordial follicle pool’s maintenance, growth, and survival [19]. AKT signaling reportedly regulates MSCs’ growth and development process [20]. However, PTEN can negatively regulate the pathway and interrupt the downstream activation of AKT [21], which then accelerates the atresia of the functional follicles and may lead to POI [22]. At the same time, down-regulation of PTEN in oocytes can activate the protein kinase B (Akt) pathway [23]. Previous investigations have indicated that the forkhead transcription factor Forkhead box O3 (FOXO3) can negatively regulate primordial follicle activation and early follicular development [24], which performs transcription of target genes when AKT is inactivated and presented as highly expressed and localized within the nucleus during the dormancy of primordial follicles. However, upon phosphorylation by AKT, FOXO3a is phosphorylated, and its transcriptional function is terminated as its localization is exported to the cytoplasm in later‑growing follicles [25], indicating that the downregulation of FOXO3a in oocytes may be a prerequisite for the initiation of oocyte growth during follicular activation in mice [26].

CD8 + CD28-T cell is an essential subset of CD8 + Tregs, which possess immunosuppressive function and participate in various inflammatory disorders and autoimmune diseases [27]. The research showed that CD8 + CD28–T cells expand after bone marrow transplantation, which may serve as a primary tolerance mechanism in transplantation and is associated with a reduced occurrence of rejection [8]. The mechanisms may be through decreasing the secretion of proinflammatory cytokines and inducing apoptosis of lymphocytes [28]. The altered expression of CD8 + CD28–T cells has been observed in cancer, viral infections, autoimmunity, and almost every chronic inflammatory disease till now [29]. However, the functional consequences of the MSC-mediated effects on CD8 + CD28-T cells should be explored further.

In this experiment, the autoimmune-induced POI mice model was built and grouped, transplanted with or without UCMSCs transfect with or without LV-hsa-miR-21/LV-hsa-miR-21-inhibition to determine whether the miR-21 expressed in UCMSCs played a critical role in the therapeutic process of POI mice with UCMSCs transplantation. To further confirm the underlying mechanisms, the expression levels of the PTEN/AKT/FOXO3a signal pathway-related cytokines in mice ovaries were detected, and the expression levels of the CD8 + CD28-T cells with the associated cytokine levels were analyzed. The research above may improve the therapeutic efficiency in treating POI patients with MSC transplantation in the clinic.

## Materials and methods

### Experimental animals

Six-week-old female mice (C57BL/6) were provided by Suzhou Zhaoyan Biotechnology Co. (Jiangsu, China). They were safely housed, fed on a standard pellet diet, and had free access to water. The Institutional Animal Care has approved all the experimental procedures and Use Committee at the First Affiliated Hospital of Soochow University. The study followed the Declaration of Helsinki and the National Research Council Guide for Care and Use of Laboratory Animals.

### Chemicals

The synthesis of the ZP3 peptide was provided by an automatic peptide synthesizer (Hangzhou Economic & Technological Development Zone, China) at 98.7% peptide purity as determined by high-performance liquid chromatography (HPLC). The amino acid composition was verified by analysis, and the amino acid sequence of the murine ZP3330-342 peptides used in this study was ASSSSGPGIHGPA.

### Isolation, culture, and identification of human UCMSCs

Human umbilical cords were obtained with written informed consent from full-term pregnant women with a negative test for HIV-I, hepatitis B, and C. All the samples were performed under standard experimental protocols the Institutional Ethics Committee permitted. The umbilical cords were first washed with phosphate-buffered saline (PBS). Then with the blood vessels removed, the tissues were cut into small pieces (1mm3) and placed onto plates treated with Low Dulbecco-modified Eagle medium (L-DMEM, Hyclone, US) supplemented with 10% fetal bovine serum (FBS, Gibco, US), 100 U/ml streptomycins and 100 U/ml penicillin G (Gibco, US) and then maintained at 370 C in a humidified atmosphere with 5% CO2. The media were renewed every 3-4d. Once reaching approximately 80–90% confluence, cells were passaged. The cell morphology was observed under an Inverted fluorescence Microscope (Nikon, Japan) to confirm the phenotype of UCMSCs. Alizarin red staining (Cyagen, US) was applied for osteogenic differentiation to identify osteoblast-like cells. Oil Red O staining (Cyagen, US) was applied for adipogenic differentiation to identify adipose cells. Additionally, the membrane and intracytoplasmic molecular markers of UCMSCs were examined using FCM. Following staining with phycoerythrin-conjugated or fluorescein isothiocyanate-conjugated mouse anti-human CD14, CD29, CD34, CD90, CD31, and HLA-DR mAb (Biolegend, US), cells were sorted with cytometry and harvested for culture [30] Cells utilized for the experiments were after three passages.

### Construction of the miR-21-5p/miR-21-5p-inhibition lentiviral vector (LV-hsa-miR-21-5p/LV-hsa-miR-21-5p-inhibition) and transfected into UCMSCs

The inhibitor/activator/empty vector of hsa-miR-21 lentivirus gene transfer vector encoding the green fluorescent protein (GFP) was constructed by Shanghai Genechem Co., Ltd. (Shanghai, China) to evaluate whether the miR-21 expressed in UCMSCs played a critical role in restoring the ovarian function of POI mice. UCMSCs were infected with lentiviral vectors at a multiplicity of infection (MOI) of 20 when grown to 20–30% confluence. The sequence of LV-hsa-miR-21-5p 5’‑CACACATTCCACAGGCTAGACCAGACAGAAGGACCAG‑3’ and LV-hsa-miR-21-5p-inhibition 5’‑CCGGTCAACATCAGTCTGATAAGCTATTTTTG‑3’ were confirmed by sequencing (data not shown). The recombinant lentivirus of hsa-miR-21-5p, hsa-miR-21-5p-inhibitor, and the control lentivirus (LV-NC, 5’-TTCTCCGAACGTGTCACGT-3’) were prepared and tittered to 1 × 108 transfection unit (TU)/ml. A total of ∽ 1 × 106 UCMSCs were plated in each well in 25cm2 plates overnight at 37˚C. Following 16–24 h of culture, lentiviruses were diluted in 2.5 ml L-DMEM (Hyclone, US) containing HitransG P and added to the cells and incubated at 37˚C for an additional 12–16 h, followed by incubation in 5 ml of fresh L-DMEM for another 72 h at 37˚C. Then, the lentivirus transduction efficiency of UCMSCs was determined by detecting GFP signals under the fluorescence microscope. Furthermore, the mir-21 levels were determined in each group using quantitative reverse-transcription PCR (QRT-PCR).

### Establishment and Grouping of Experimental Mice Models

Adult mice (*n* = 54) were randomly divided into six groups (*n* = 9): control group (A), POI group (B), POI + UCMSCs group (C), POI + LV-NC-UCMSCs group (D), POI + LV-hsa-miR-21-UCMSCs group (E), POI + LV-hsa-miR-21-inhibition-UCMSCs group (F). Mice in group A received no treatments. Mice in group B-F were first injected i.h. Subcutaneously with 50 nmol/L of ZP3 (mouse) emulsified in complete Freund’s adjuvant (CFA) (Mycobacterium tuberculosis H37RA strain, 0.16 mg/mouse; Sigma) one week after adaptive feeding, and then injected with 50 nmol/L of ZP3 (mouse) emulsified in Freund’s incomplete adjuvant (FIA) (M. tuberculosis H37RA strain, 0.16 mg/mouse; Sigma) two weeks later [1]. Then, one week later, the cell suspension containing 1 × 10^6^ UCMSCs of sixth passages with PBS was injected into mice in group C. According to the previous studies, equal amounts of cell suspension containing UCMSCs transfected with LV-NC/LV-hsa-miR-21/LV-hsa-miR-21-inhibition were separately injected into mice in groups D, E, and F [31]. One week later, all mice were sacrificed to do the following experiments.

### Serum levels of hormone, AZPAb, and Interleukin (IL-10) measurement

All mice blood samples were obtained from postcava and centrifuged at 4000 r/min for 10 min to get the mice serum. The levels of AZPAb, IL-10, and the levels of hormones, including estradiol (E2), follicle stimulation hormone (FSH), luteinizing hormone (LH), anti-Müllerian hormone (AMH), and IL-10 were quantified by ELISA kits (Greenleaf, CN) according to manufacturer’s instructions.

### Ovarian follicle counting and morphological analysis

Mice ovaries were collected, washed, fixed, and stained with HE for histopathology. The ovarian histological examination was performed using light microscopy (Olympus). The follicles were counted only on those containing an oocyte with a visible nucleus. According to the previously described method, the follicles were detected and classified as primordial, primary, secondary, antral, and atretic follicles [32].

### QRT-PCR

According to the manufacturer’s protocols, total RNA from mice ovaries was extracted using the RNeasy Mini Kits (Qiagen, Germany). Amounts of 1 µg of total RNA were subjected to reverse mRNA transcription using oligo dT as a primer and a reverse transcription kit (Transgene Biotech, China) to generate total cDNA. The quantitative PCR was then carried out using primers shown in Table 1 and FastStart Universal SYBR Green Master (Thermo Fisher Scientific, US) with the StepOnePlusTM Real-Time PCR System (Thermo Fisher Scientific, US). GAPDH was used for normalization. For analysis of mir-21 expression in ovaries, Rnu6 (U6) was used to normalize data due to an equivalent size. Mir-21 was quantified using the TaqMan® MicroRNA Reverse Transcription kit (Applied Biosystems Carlsbad, CA) for the reverse transcription (RT) reaction, and the primers and probe used were TaqMan® MicroRNA Assay for LV-hsa-miR-21-5p/LV-hsa-miR-21-5p-inhibition (Applied Biosystems, Carlsbad, CA) according to manufacturer’s recommendations. Each sample in each group was detected in triplicate.

**Table 1:** Sequences of the primers used in QRT-PCR.

Target gene	Primer	Nucleotide sequence
*h-Rnu6(U6)*	F	5’-TGCGGGTGCTCGCTTCGGCAGC-3’
	R	5’-CCAGTGCAGGGTCCGAGGTA-3’
*h-miR-21*	F	5’-TAGCTTATCAGACTGATGTTG-3’
	R	5’-GCTGTCAACGATACGCTACGTAACG-3’
*mus-Rnu6(U6)*	F R	5’-CTCGCTTCGGCAGCACA-3’ 5’-AACGCTTCACGAATTTGCGT-3’
*mus-miR-21*	F R	5’-ACACTCCAGCTGGGTAGCTTATCAGACTGATG-3’ 5’-CTTAACGGCTGAGGTGCTGT-3’
*mus-priMir21*	F R	5’-GACATCGCATGGCTGTACCA-3’ 5’-CCATGATTCAACAGTCAACATCA-3’
*mus-GAPDH*	F	5’-AGGTCGGTGAACGGATTTG-3’
	R	5’-TGTAGACCATGTAGTTGAGGTCA-3’
*mus-PTEN*	F	5’-TGGATTCGACTTAGACTTGACCT-3’
	R	5’-TGGCGGTGTCATAATGTCTCT-3’
*mus-FOXO3a*	F	5’-CTGGGGGAACCTGTCCTATG-3’
	R	5’-TCATTCTGAACGCGCATGAAG-3’
*mus-caspase3*	F	5’-TGGTGATGAAGGGGTCATTTATG − 3’
	R	5’-TTCGGCTTTCCAGTCAGACTC-3’

### Western blotting

Ovaries were lysed by radioimmunoprecipitation assay (RIPA) buffer, and the protein concentration was measured by bicinchoninic acid assay. Proteins were separated using 10% sodium dodecyl sulfate-polyacrylamide gel electrophoresis (SDS-PAGE) gel electrophoresis and transferred to a polyvinylidene difluoride (PVDF) membrane. The membrane was blocked with 6% skim milk powder at room temperature, followed by the incubation of primary antibodies against PTEN (1:1000, Proteintech), p-PTEN(Ser380)(1:1000, CST), AKT (1:5000, Proteintech), p-AKT(Ser473)(1:1000, CST), FOXO3a (1:1000, Abbkine), p-FOXO3a (Ser253)(1:1000, Abbkine), caspase3 (1:1000, CST), and GAPDH (1:30000, Proteintech) overnight at 40 C. After washing the membranes three times, the secondary antibodies were incubated for one hour at room temperature. Protein expression was detected using the Super Enhancer chemiluminescence (ECL) Kit (Absin, CN), and band intensity was quantified using Image J software.

### Immunohistochemistry

Mice ovaries were washed, fixed, and cut into Sect. (4 μm). The microwave method was used for antigen retrieval. After the adaption of H2O2 and blocked with the goat serum, the sections were then incubated with rabbit primary polyclonal antibodies against mouse PTEN (1:200, CST), p-PTEN (Ser380) (1:50, CST), p-AKT (Ser473) (1:100, CST), FOXO3a (1:200, Abbkine), and p-FOXO3a (Ser253) (1:200, Abbkine) at 4 °C overnight. Then, they were incubated with biotinylated secondary antibodies at 37 °C for 30 min. The reaction products were developed with diaminobenzidine (DAB) as chromogen and counterstained with hematoxylin. The staining results were scored using the German immunoreactive score (IRS). The staining intensity was graded as “0” (negative), “1” (weak), “2” (moderate), and “3” (strong); the staining extent was graded as “0” (< 5%), “1” (5–25%), “2” (25–50%), “3” (50–75%) or “4” (> 75%). The staining intensity values and the staining extent were multiplied as a final IRS.

### Differentiation of CD8^+^CD28^−^T lymphocytes by flow cytometry (FCM)

To determine the expression of CD8 + CD28-T cells in mice, FCM analysis was performed on isolated spleen cells using anti-mouse CD3, CD8, and CD28 monoclonal antibodies. The spleens were minced mechanically and lysed in a lymphocyte separation medium. The isolated spleen cells were washed and resuspended in PBS. Anti-mouse CD3 APC, anti-mouse CD8 FITC, and anti-mouse CD28 PE (eBioscience, San Diego, USA) were mixed at 4 0 C for 10 min in the dark. And then, the cell suspension was analyzed using FCM.

### Data analysis

Analyses were performed using SPSS 16.0 software. Each experiment was performed at least thrice, and the continuous experimental data are shown as the mean ± standard deviation. Results were analyzed statistically using Student’s t-test for comparisons between two groups. A one-way analysis of variance (ANOVA) was used to distribute data. A P-value of < 0.05 was considered statistically significant.

## Results

### The Primary Culture of UCMSCs and the Transfection Efficiency of UCMSCs with LV-hsa-miR-21-5p/LV-hsa-miR-21-5p-inhibition

**Fig. 1:**
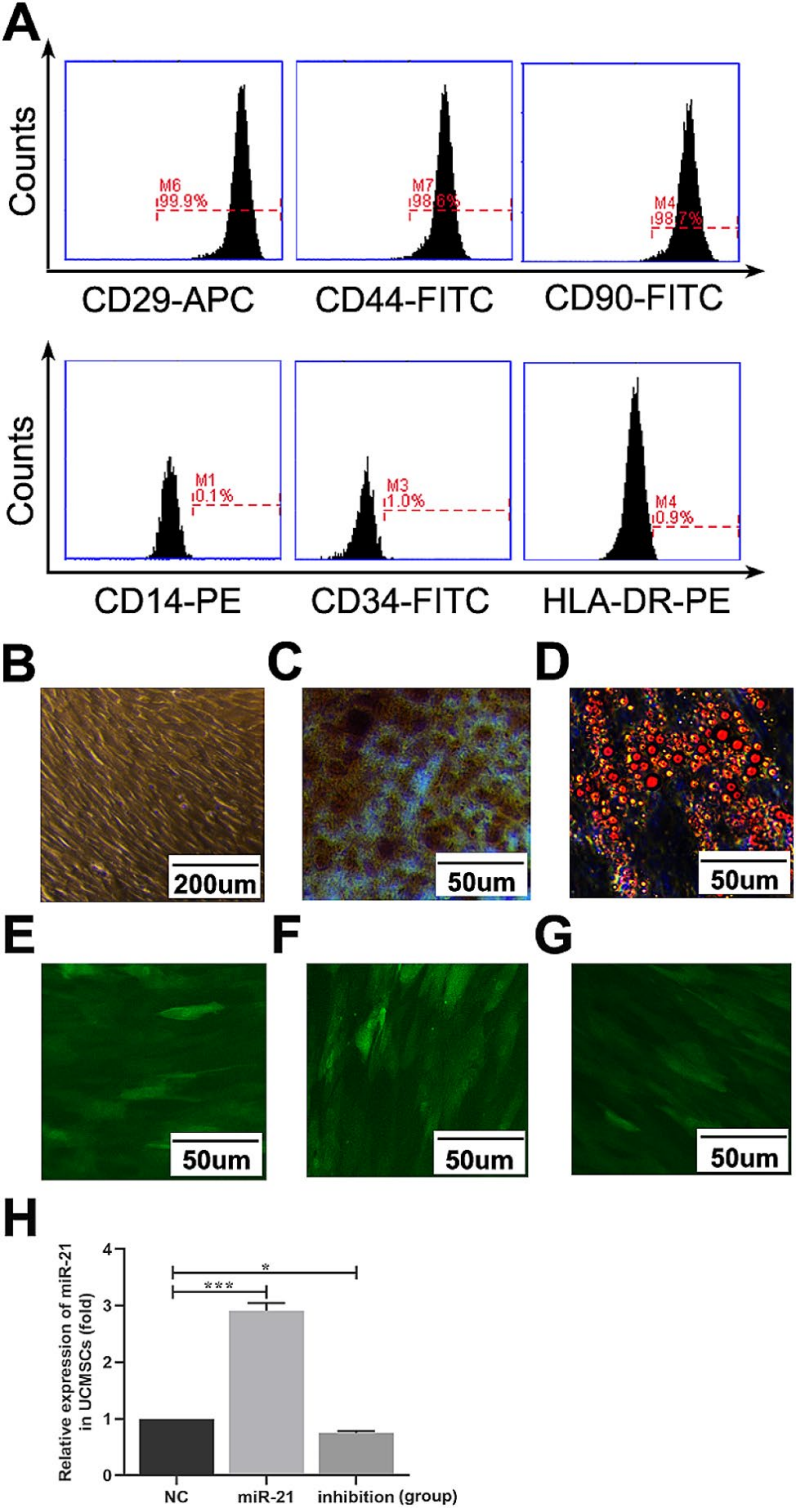
Identification of UCMSCs, and the transfection efficiency of the LV-hsa-miR-21-5p/LV-hsa-miR-21-5p-inhibition into UCMSCs. **A** Black histograms represent the expression of UCMSCs specific surface marker. **B** The cultured UCMSCs present as the fibroblast-like morphology. **C** and **D** UCMSCs differentiated into osteoblasts or lipoblasts in specific conditions. Osteoblasts are displayed by Alizarin Red staining and darker red staining indicates calcium deposition (**C**). Lipoblasts were displayed by the accumulation of neutral lipid vacuoles stained with Oil Red O (**D**). **E** and **F** The GFP staining of miR-21 in UCMSCs transfected with LV-hsa-miR-21-5p/LV-hsa-miR-21-5p-inhibition/LV-NC. **G** The transfection efficiency of the LV-hsa-miR-21-5p/LV-hsa-miR-21-5p-inhibition into UCMSCs. ^*^P < 0.05, ^***^P < 0.001 vs. LV-NC-UCMSCs group

After 7–10 days of inoculation, cells began to crawl out of the tissue and form individual clone spheres, presented as fibroblast-like morphology (Fig. 1B). A homogenous cell population can be observed three passages later, and the morphology can be stable for up to 10 passages. The immunophenotyping analysis showed positive expression of mesenchymal progenitor markers with CD29, CD44, and CD90. The hematopoietic cell surface markers of CD34, CD14, and HLA-DR are negative (Fig. 1A). In an in vitro conditional culture system, UCMSCs were induced to develop into different lineages. In the osteoblastic induction medium, von Kossa staining showed calcium deposition (Fig. 1C). Fat globules were present in the cytoplasm in the adipogenic induction medium, and Oil Red O staining was positive (Fig. 1D). Results are consistent with the previous literature report [30].

UCMSCs were transfected with LV-hsa-miR-21/LV-hsa-miR-21-inhibition/LV-NC at an MOI of 20, and the expression of GFP-marked lentivirus in each group was observed under the fluorescence microscope (Fig. 1(E-G)). The transfection efficiency was ≥ 90%. The results of QRT-PCR further indicated that a significantly higher expression level of miR-21 was observed in the LV-hsa-miR-21 group (*P* < 0.001). Lower expression levels were observed in the LV-hsa-miR-21-inhibition group compared with the LV-NC group (*P* < 0.05) (Fig. 1H), which represents that the LV-hsa-miR-21/LV-hsa-miR-21-inhibition has been effectively transfected into UCMSCs, and the role that miR-21 played was correspondently enhanced/weakened.

### MiR-21 Expressed in UCMSCs Played a Critical Role in Recovering the Ovarian Function in POI Mice

To evaluate the critical role of the miR-21 expressed in UCMSCs during the therapeutic process, the POI mice were transplanted with LV-hsa-miR-21-UCMSCs/LV-hsa-miR-21-inhibition-UCMSCs to evaluate the changes in the ovarian function. The transcription of the miR-21 in different groups of mice was first analyzed by detecting the expression of pri-miR-21 and miR-21. Mice ovaries in the LV-hsa-miR-21-UCMSCs group exhibited an increased expression of pri-miR-21 transcript (*P* < 0.01), while a decreased expression was analyzed in the LV-hsa-miR-21-inhibition-UCMSCs group (*P* < 0.01) (Fig. 4A). The mature miR-21 expression followed pri-miR-21 expression and showed levels inclined in the LV-hsa-miR-21-UCMSCs group (*P* < 0.001) but declined in the LV-hsa-miR-21-inhibition-UCMSCs group (*P* < 0.05) (Fig. 4B). The results above suggested that the UCMSCs transfected with the LV-hsa-miR-21/LV-hsa-miR-21-inhibition have been successfully transplanted into the POI mice.

**Fig. 2:**
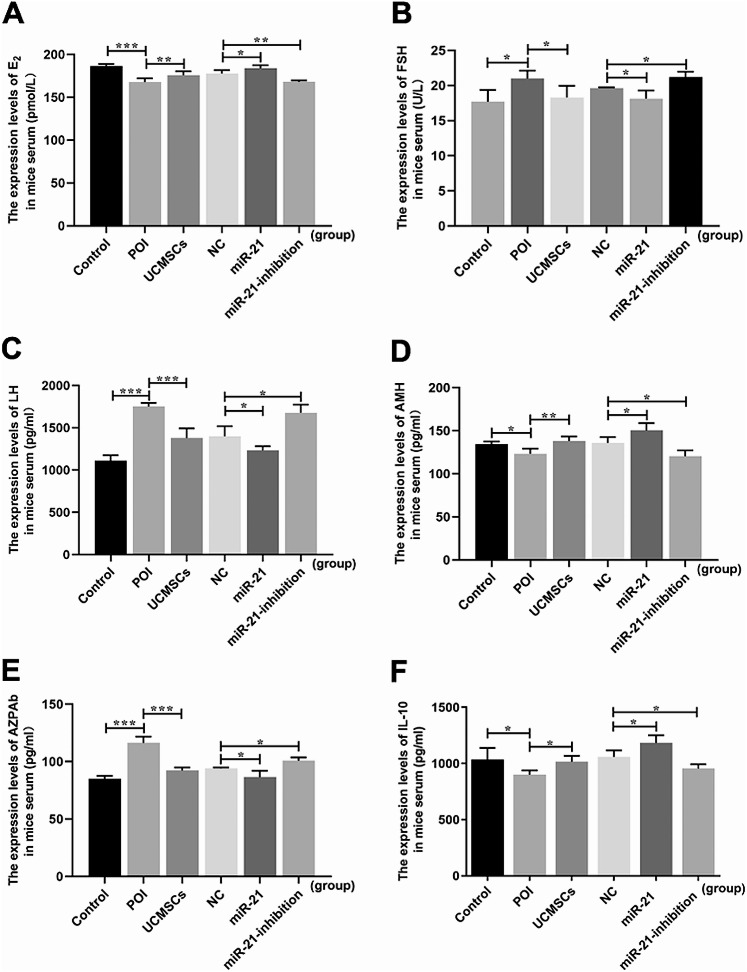
Serum levels of E_2_, FSH, LH, AMH, AZPAb, and IL-10 in mice. **A** E_2_ release. **B** FSH release. **C** LH release. **D** AMH release. **E** AZPAb release. **F** IL-10 release Data presented as mean ± SD. ^*^P < 0.05, ^**^P < 0.01, ^***^P < 0.001 vs. Control group or LV-NC-UCMSCs group, separately

The mice’s serum levels of the AZPAb and the hormone, the ovarian morphological changes, folliculogenesis, and the apoptosis of ovarian cells were measured to evaluate ovarian function. For the detection of the serum levels of AZPAb, it was inclined in the POI group (*P* < 0.001) but declined in mice with UCMSCs transplantation(*P* < 0.001). Furthermore, the levels can be down-regulated in mice with LV-hsa-miR-21-UCMSCs transplantation (*P* < 0.05). In contrast, the up-regulation levels were observed in the LV-hsa-miR-21-inhibition-UCMSCs group compared with that in the LV-NC group (*P* < 0.05)(Fig. 2E). For the evaluation of the hormone levels in mice serum, decreased levels of E2 (*P* < 0.001) and AMH (*P* < 0.05) were detected, with increased levels of FSH (*P* < 0.05) and LH (*P* < 0.001) in the POI group comparing with the control group. At the same time, the transplantation of UCMSCs can reverse the changes above, shown as increased levels of E2 (*P* < 0.01) and AMH (*P* < 0.01), with decreased levels of FSH (*P* < 0.05) and LH (*P* < 0.001). Moreover, the uptrend levels of E2 (*P* < 0.05) and AMH (*P* < 0.05), with the downtrend levels of FSH (*P* < 0.05) and LH (*P* < 0.05), were observed in the LV-hsa-miR-21-UCMSCs group. In contrast, the downtrend levels of E2 (*P* < 0.01) and AMH (*P* < 0.05), with the uptrend levels of FSH (*P* < 0.05) and LH (*P* < 0.05) were detected in the LV-hsa-miR-21-inhibition-UCMSCs group compared with that in the LV-NC-UCMSCs group (Fig. 2(A-D)), from which we can primarily affirm the critical role of the miR-21 in POI mice with UCMSCs transplantation.

**Fig. 3:**
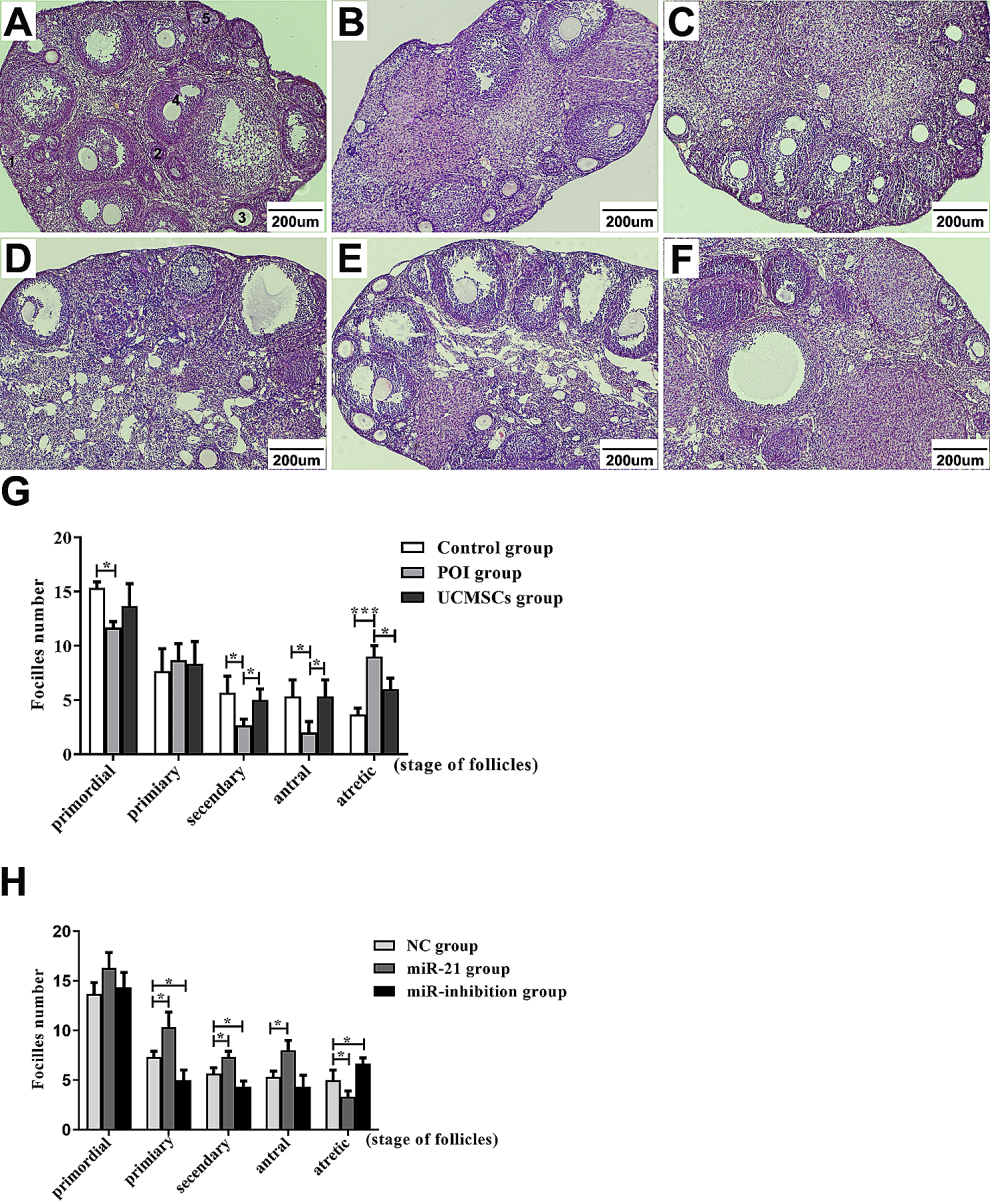
Histopathological examination of mice ovarian tissues. Photomicrographs shows H&E stained ovaries. **A** Control group. **B** POI group. **C** UCMSCs group. **D** LV-NC-UCMSCs group. **E** LV-hsa-miR-21-5p-UCMSCs group. **F** LV-hsa-miR-21-5p-inhibition-UCMSCs group. Five types of ovarian follicles were marked in each group (1–5). **G** and **H** Statistical charts of the follicle count in mice ovaries of the six groups. Data presented as mean ± SD. ^*^P < 0.05, ^**^P < 0.01, ^***^P < 0.001 vs. Control group or LV-NC-UCMSCs group, separately. Bar scale = 200 μm

**Fig. 4:**
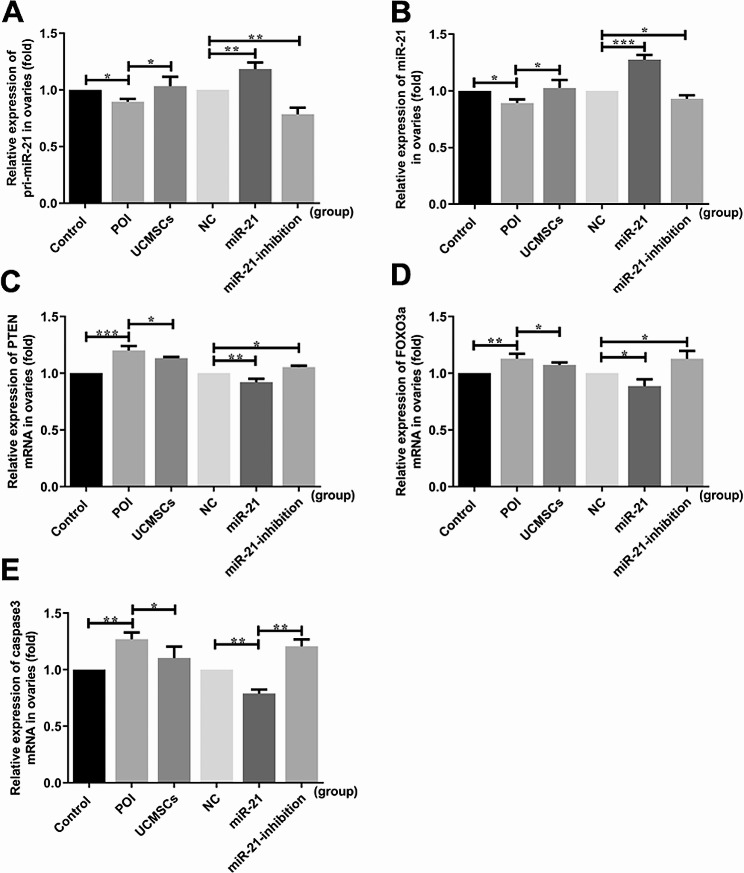
The mRNA level changes in mice ovaries of the associated cytokines in different groups. **A** Pri-miR-21 expression. **B** MiR-21 expression. **C** PTEN mRNA expression. **D** FOXO3a mRNA expression. **E** Caspase 3 mRNA expression. ^*^P < 0.05, ^**^P < 0.01, ^***^P < 0.001 vs. Control group or LV-NC-UCMSCs group, separately

**Fig. 5:**
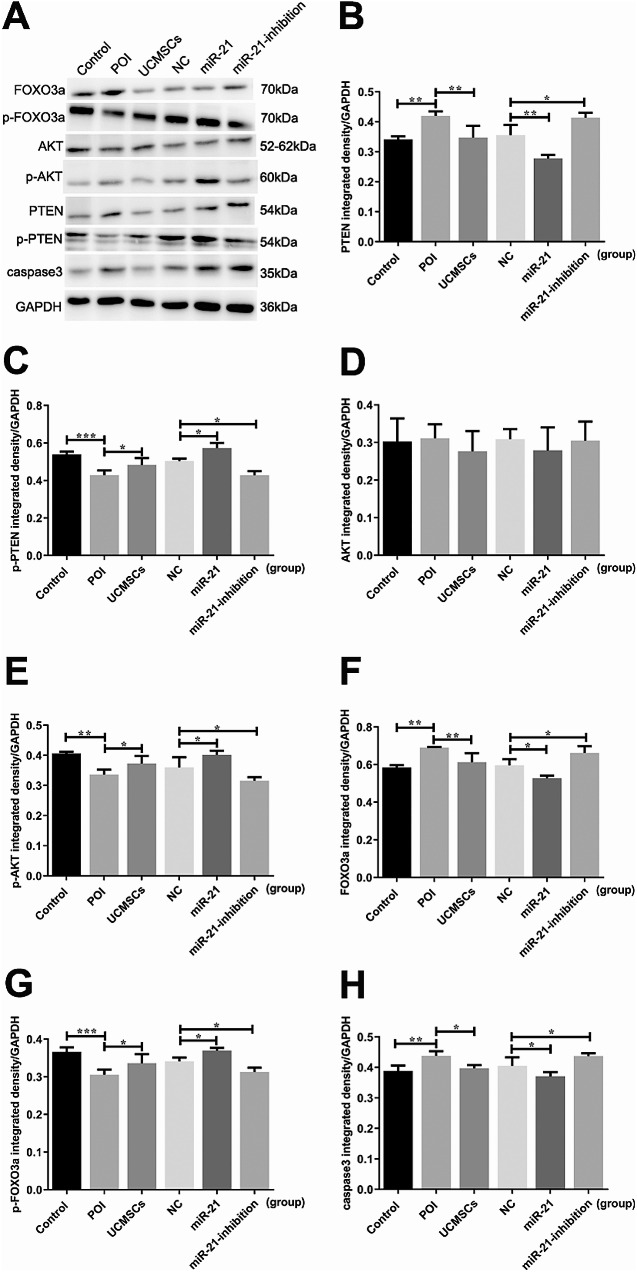
The protein levels in mice ovaries of the associated cytokines with different treatment. **A** The caspase 3 and the PTEN/AKT/FOXO3a signal pathway-related protein expression in PVDF membrane. Quantification of **B** PTEN, **C** p-PTEN, **D** AKT, **E** p-AKT, **F** FOXO3a, **G** p-FOXO3a, and **H** caspase 3 protein expression. ^*^P < 0.05, ^**^P < 0.01, ^***^P < 0.001 vs. Control group or LV-NC-UCMSCs group, separately

For the aspect of the ovarian morphology, different stages of follicles, including primordial follicles (Fig. 3A − 1), primary follicles (Fig. 3A − 2), secondary follicles (Fig. 3A− 3), antral follicles (Fig. 3A-4), and atretic follicles (Fig. 3A-5) were observed in mice ovaries (Fig. 3). In the POI group, the morphology of the ovarian tissues presented as fibrosis-like, with a decreased number of primordial follicles (*P* < 0.05), secondary follicles (*P* < 0.05), and antral follicles (*P* < 0.05), and an increased number of atretic follicles compared with the control group (*P* < 0.001)(Fig. 3B). With the UCMSCs transplantation, the damages above can be partly reversed, which is shown as the reduced degree of ovarian fibrosis, with the increased number of secondary follicles (*P* < 0.05) and antral follicles (*P* < 0.05) and the decreased number of the atretic follicles (*P* < 0.05)(Fig. 3C). Additionally, it presented as the atrophied ovaries in the LV-hsa-miR-21-inhibition-UCMSCs group, which were mainly composed of interstitial cells in a fibrous matrix (Fig. 3F), with a declined number of primary follicles (*P* < 0.05) and secondary follicles (*P* < 0.05), but an inclined number of atretic follicles (*P* < 0.05) when comparing with the LV-NC-UCMSCs group. However, in the LV-hsa-miR-21-UCMSCs group, the degree of ovarian fibrosis was reduced (Fig. 3E), with an inclined trend of primary follicles (*P* < 0.05), secondary follicles (*P* < 0.05), and antral follicles (*P* < 0.05), but a declined trend of atretic follicles (*P* < 0.05).

Additionally, the mRNA and protein levels of caspase 3 were measured to evaluate the cells’ apoptosis in mice ovaries. The caspase 3 mRNA levels were detected up-regulated in the POI group (*P* < 0.01) compared with the control group, while it was down-regulated in mice with UCMSCs transplantation (*P* < 0.05). In mice with LV-hsa-miR-21-inhibition-UCMSCs transplantation, the inclined trend of the caspase 3 mRNA levels was observed (*P* < 0.01). However, the LV-hsa-miR-21-UCMSCs transplantation can alleviate the apoptosis showed as the down-regulated levels of caspase 3 expression (*P* < 0.01)(Fig. 4E). Correspondingly, compared with the LV-NC group, the protein levels of caspase 3 in the miR-21-inhibition group were similar to the POI group presented on the inclined curve (*P* < 0.05). At the same time, it declined in the LV-hsa-miR-21-UCMSCs group (*P* < 0.05)(Fig. 5H). Based on the results above, we can further confirm that the miR-21 played a critical role during the therapeutic process of POI mice receiving UCMSCs transplantation.

### MiR-21 Expressed in UCMSCs Restore the Ovarian Function of POI Mice through Inhibiting the PTEN/AKT/FOXO3a Signal Pathway

To investigate the molecular mechanisms of the miR-21 expressed in UCMSCs in restoring mice’s ovarian function with POI disorder, the PTEN/AKT/FOXO3a signal pathway-related mRNA and protein levels were analyzed. Results showed that the decreased levels of the PTEN and FOXO3a mRNA were analyzed in the LV-hsa-miR-21-UCMSCs groups compared with that in the LV-NC group (*P* < 0.01&*P* < 0.05), while in the LV-hsa-miR-21-inhibition-UCMSCs group, it presented as the inclined trend of the PTEN and FOXO3a mRNA levels (*P* < 0.05& *P* < 0.05) (Fig. 4C and D). No significant differences between the groups were observed in AKT mRNA expression (results not shown in the picture). For the aspect of protein expression, decreased protein levels of PTEN (*P* < 0.01)(Fig. 5B) and FOXO3a (*P* < 0.05)(Fig. 5F) were detected in the LV-hsa-miR-21-UCMSCs group, and the increased levels of p-PTEN (*P* < 0.05)(Fig. 5C), p-FOXO3a (*P* < 0.05)(Fig. 5G), and p-AKT (*P* < 0.05)(Fig. 5E) were detected. However, in mice with the LV-hsa-miR-21-inhibition-UCMSCs transplantation, up-regulated levels of PTEN (*P* < 0.05) and FOXO3a (*P* < 0.05) were observed, accompanied by the down-regulated levels of p-PTEN, p-AKT, and p-FOXO3a (*P* < 0.05)(Fig. 5). From the detections above, it is confirmed that the miR-21 expressed in UCMSCs played its role by negatively regulating the PTEN/AKT/FOXO3a signal pathway by phosphorylating each cytokine.

**Fig. 6:**
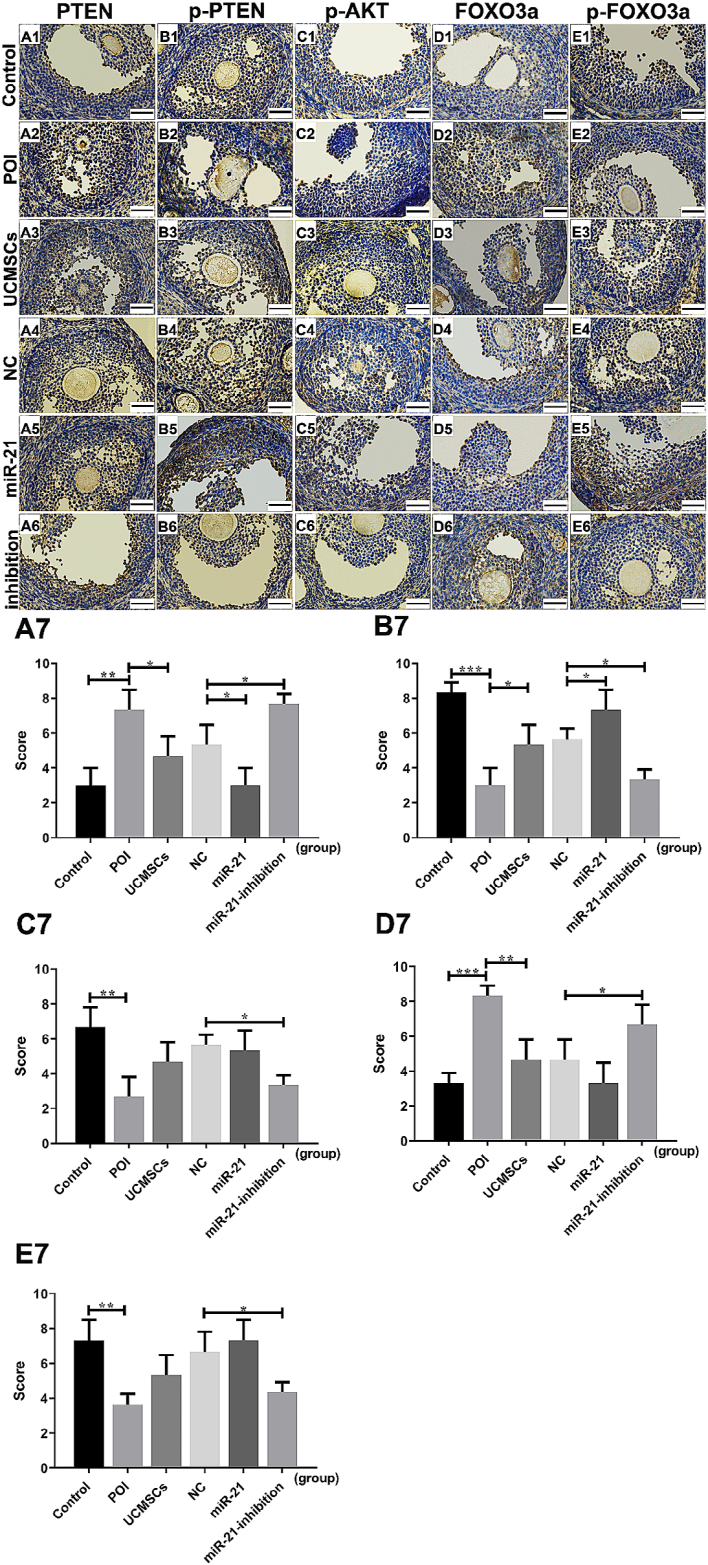
IHC analysis on PTEN, p-PTEN, p-AKT, FOXO3a, and p-FOXO3a in ovarian tissue of mice. Photomicrographs show hematoxylin and DAB-stained ovaries. (A1-E1) Control group. (A2-E2) POI group. (A3-E3) POI + UCMSCs group. (A4-E4) POI + LV-NC-UCMSCs group, (A5-E5) POI + LV-hsa-miR-21-UCMSCs group, (A6-E6) POI + LV-hsa-miR-21-inhibition-UCMSCs group. The statistical charts of the five kinds of cytokines expression in the six groups (A7-E7). Brown in cytoplasm indicates positive expression of the aimed protein. Blue represents cell nuclear staining. ^*^P < 0.05, ^**^P < 0.01, ^***^P < 0.001 vs. Control group or LV-NC-UCMSCs group, separately. Bar scale = 50 μm

To further confirm and localize the changes of the PTEN/AKT/FOXO3a signal pathway-related cytokines in the ovaries, the IHC analysis was used in the experiment. In the POI group, the PTEN and FOXO3a levels in mice ovaries were increased (*P* < 0.01&*P* < 0.001) with the decreased levels of p-PTEN, p-AKT, and p-FOXO3a (*P* < 0.001&*P* < 0.01&*P* < 0.01); At the same time, the UCMSCs transplantation decreased the expression of the PTEN and FOXO3a compared with the control group(*P* < 0.05&*P* < 0.01) but increased the levels of p-PTEN (*P* < 0.05). Results showed that the levels of PTEN declined (*P* < 0.05) in the LV-hsa-miR-21-UCMSCs group, with inclined levels of p-PTEN (*P* < 0.05). While in mice with LV-hsa-miR-21-inhibition-UCMSCs treatment, the expression levels of PTEN and FOXO3a were increased (*P* < 0.05), with decreased levels of p-PTEN, p-AKT, and p-FOXO3a (*P* < 0.05)(Fig. 6). Taken together, we can verify that the PTEN/AKT/FOXO3a signal pathway changes localized in mice ovaries, which is vital in the recovery function of mice with LV-hsa-miR-21-UCMSCs transplantation.

### MiR-21-UCMSCs transplantation up-regulates the frequency of CD8^**+**^**CD28**^**–**^T cells in POI mice

**Fig. 7:**
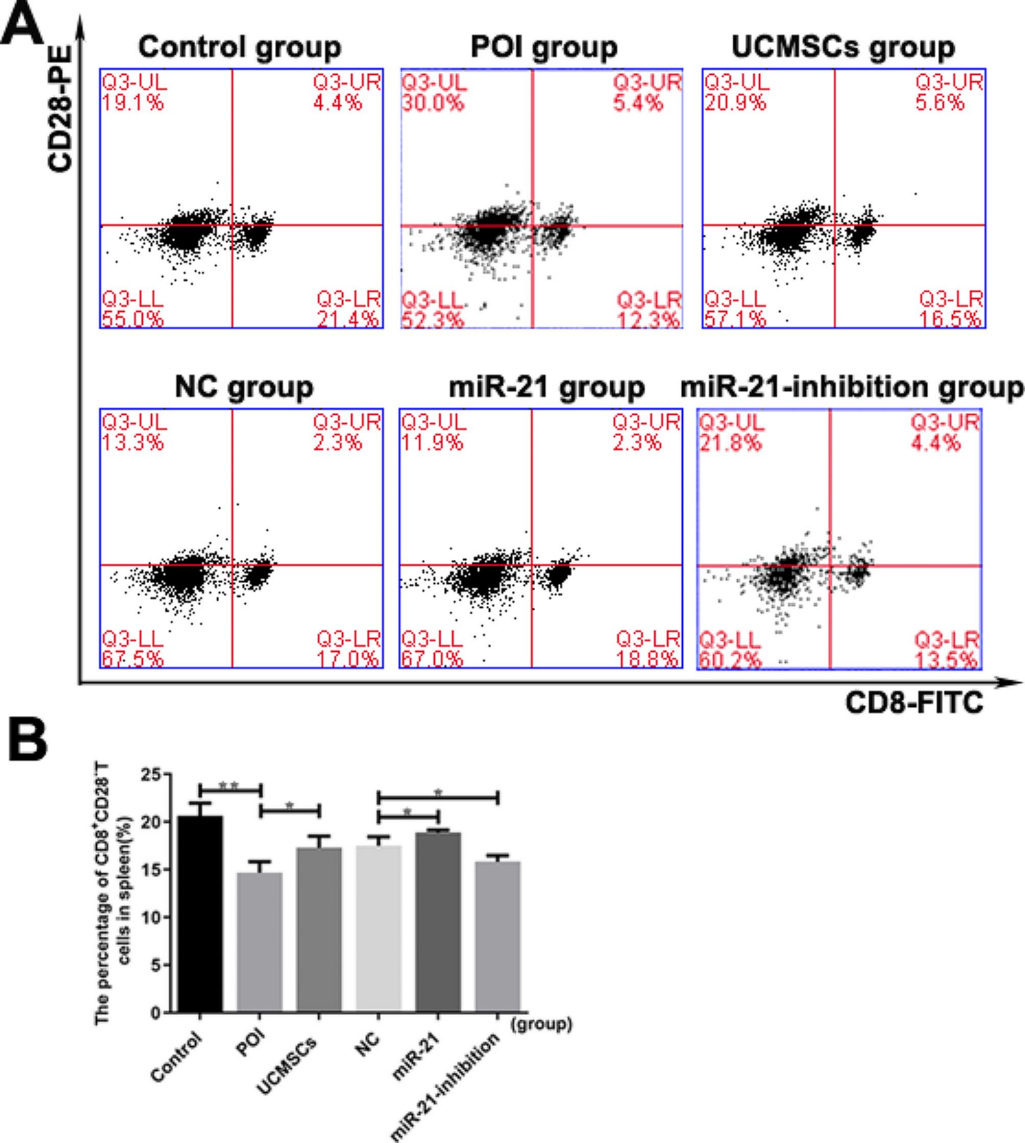
The expression levels of the CD8^+^CD28^−^T cells in different group of mice. **A** Representative flow cytometric plots for CD8^+^CD28^−^T acquisition isolated from mice spleens. **B** The statistical chart of the CD8^+^CD28^−^T cells population in the six groups. ^*^P < 0.05, ^**^P < 0.01 vs. Control group or LV-NC-UCMSCs group, separately

To assess the effects of the miR-21 on POI mice’s therapeutic effect with UCMSCs transplantation to the proportion of CD8 + CD28–T cells, spleen cells were analyzed in autoimmune-induced POI mice transplanted UCMSCs transfected with or without LV-hsa-miR-21/ LV-hsa-miR-21-inhibition (Fig. 7A). Results showed that compared with the LV-NC group, the frequency of CD8 + CD28–T cells was higher in the LV-hsa-miR-21-UCMSCs group (*P* < 0.01) but lower in the LV-hsa-miR-21-inhibition-UCMSCs group (*P* < 0.05), which means that the miR-21 can promote the therapeutic efficiency of UCMSCs through promoting the expression of CD8 + CD28-T cells (Fig. 7B). IL-10 is one of the critical predictors of inflammation produced by CD8 + CD28-T cells, which can be further detected to confirm the expression of CD8 + CD28-T cells during the therapeutic process. As shown in Fig. 2F, with the LV-hsa-miR-21-UCMSCs transplantation, the serum levels of IL-10 were elevated (*P* < 0.05), however, the expression was reduced in mice with LV-hsa-miR-21-inhibition-UCMSCs transplantation (*P* < 0.05). Taken together, we can conclude that the miR-21 may improve the therapeutic role of UCMSCs by increasing the circulating CD8 + CD28-T Cells.

## Discussion

MSC transplantation has been recognized as a promising treatment for POI disorder in animal models [33]. The UCMSCs have been chosen as the most appropriate cells for clinical applications because of their easy accessibility, immunoregulatory effect, and low immunogenicity [34]. However, the clinical therapeutic effect still needs to be elevated, and the complicated mechanism remains to be explored. Previous studies have proved that MSCs can migrate to the injured ovaries following intravenous transplantation [35, 36]. In our experiments, we are mainly attributed to MSC-derived biological factors, anti-inflammatory and immunosuppressive capabilities to repair ovarian dysfunction, not “homing” effect, so we did not prove the localization of UCMSCs in mice ovaries. In this study, the POI mice model was built up with the characteristics of the abnormally elevated serum levels of AZPAb and the disordered hormone, including the increased levels of FSH and LH and the decreased levels of E2 and AMH; the atrophied ovaries with the enhanced degree of fibrosis, and the decreased number of the functional follicles and the increased number of the atretic follicles; besides, the cells’ apoptosis in mice ovaries were increased showed as the higher expression levels of the caspase3 mRNA and protein. We also proved that the injured ovarian function could be recovered mainly in POI mice with UCMSCs transplantation, which presented as the restored serum levels of AZPAb and the hormone, including the up-regulated levels of E2 and AMH, and the down-regulated levels of LH and FSH (Fig. 2); the ovarian morphology can be improved with decreased levels of atrophy and fibrosis, and the number of functional follicles increased, and atretic follicles decreased (Fig. 3); moreover, the decreased levels of the ovarian cells’ apoptosis were observed presented as the decreased levels of caspase3 mRNA and protein (Figs. 4F and 5H). Unfortunately, differences still exist between the control group and the UCMSCs group, so one of the aims of the study is to improve the therapeutic effect of UCMSCs transplantation.

Pieces of evidence have demonstrated that miR-21 plays a regulatory role in ovarian granulosa cell apoptosis and follicular development [37]. However, it is still unknown whether the miR-21 expressed in UCMSCs is essential to recovering the POI mice’s ovarian function. This study transplanted UCMSCs transfected with or without the LV-hsa-miR-21/LV-hsa-miR-21-inhibition to treat autoimmune-induced POI mice. The up-regulated pri-mir-21 and mir-21 mRNA levels in the mir-21 group and down-regulated levels in the miR-21-inhibition group testified the successful transfection of LV-hsa-miR-21/miR-21-inhibition into UCMSCs (Fig. 4A and B). By comparing the mice’s outcomes with LV-hsa-miR-21/LV-hsa-miR-21-inhibition-UCMSCs transplantation, we have confirmed that the mice’s ovarian function recovery is essentially associated with the presence/absence of the miR-21. The absence of miR-21 in UCMSCs transplantation decreased the levels of E2 and AMH. However, it increased the levels of FSH and LH in mice serum (Fig. 2). The enhanced ovarian fibrosis with the decreased number of functional follicles and the increased number of atretic follicles were observed in mice ovaries (Fig. 3) along with the increased levels of the ovarian cells’ apoptosis (Figs. 4 and 5). However, the presence of miR-21 in UCMSCs transplantation strikingly recovers the ovarian function of POI mice with all these indices improved, which showed with the increased levels of E2 and AMH (Fig. 2), but decreased levels of FSH and LH in mice serum, and the released levels of ovarian fibrosis, with decreased functional follicles in mice ovaries (Fig. 3). Also, there are decreased levels of ovarian cell apoptosis. Based on this evidence, we can primarily recognize that miR-21 expressed in UCMSCs played a critical therapeutic role in recovering the POI mice’s ovarian function.

The involved mechanisms are explored in this study to understand better the role of miR-21 in the therapeutic process of POI mice with UCMSCs transplantation. PTEN is a confirmed downstream target gene of miR-21 [38], an antagonist of the PI3K/AKT signal [39]. To verify whether the PTEN/AKT/FOXO3a signal pathway participates in the therapeutic process of POI mice with UCMSCs transplantation with/without the transfection of the LV-hsa-miR-21-5p/LV-hsa-miR-21-5p-inhibition, the associated mRNA and protein levels of the pathway were analyzed in this study. It is revealed that the PTEN/AKT/FOXO3a signal pathway was activated in mice with the LV-hsa-miR-21-5p-inhibition-UCMSCs transplantation, presented as the up-regulated levels of PTEN and FOXO3a, with the decreased levels of the p-PTEN, p-AKT, and p-FOXO3a. Adversely, in the LV-hsa-miR-21-5p-UCMSCs group, the signal pathway was inhibited, which showed the down-regulated mRNA and protein levels of PTEN and FOXO3a mRNA, along with the increased protein levels of the p-PTEN, p-AKT, and p-FOXO3a. From the results above, we can conclude that the miR-21 improves the ovarian function recovery of POI mice with UCMSCs transplantation, which can be associated with the inhibition of the PTEN/AKT/FOXO3a signal pathway in mice ovaries.

Like traditional regulatory T cells, CD8 + CD28–T cells possess pleiotropic immunosuppressive effects [40]. Results in our study showed that the circulating CD8 + CD28–T cells inclined in mice with LV-hsa-miR-21-UCMSCs transplantation compared with that in the LV-NC-UCMSCs group but declined in mice with LV-hsa-miR-21-inhibition-UCMSCs transplantation, suggesting that the miR-21 plays a critical role in the therapeutic process and a high level of CD8 + CD28- T cells is favorable for improving the therapeutic efficiency. To further confirm the results above, the serum levels of IL-10 in different mice groups were analyzed. IL-10 is one of the critical anti-inflammatory cytokines, contributing to CD8 + CD28–T cells’ ability to regulate T-cell responses [41]. The inclined levels of IL-10 in the LV-hsa-miR-21-UCMSCs group and declined levels in the LV-hsa-miR-21-inhibition-UCMSCs group further indicate that the immunological mechanism involved in the therapeutic process may have a close relationship with the expression changes of CD8 + CD28-T cells and its secreted anti-inflammatory cytokines.

In summary, we have shown that with the UCMSCs transplantation, the injured ovarian function of POI mice can be partially recovered, but the therapeutic efficiency needs to be enhanced. Moreover, the miR-21 can improve the recovery of ovarian function in POI mice with UCMSCs transplantation, and its mechanisms have a close relationship with the suppression of the PTEN/AKT/FOXO3a signal pathway. Furthermore, the immunological mechanism involved may be connected with the inclined levels of the CD8^+^CD28^−^T cells and the secreted anti-inflammatory cytokines. These findings may provide a new target for the subsequent MSC-based therapies in humans in clinical.
